# Reciprocal Dynamics of Therapeutic Alliance and Depressive Symptoms in Inpatient Cognitive Behavioural Analysis System of Psychotherapy: The Role of Attachment Insecurity

**DOI:** 10.1002/cpp.70200

**Published:** 2025-12-08

**Authors:** Mona L. Diehm, Julia I. Kunz, Stephan Goerigk, Johannes Wolf, Jennifer Lange, Andrea Jobst, Frank Padberg, Matthias A. Reinhard

**Affiliations:** ^1^ Department of Psychiatry and Psychotherapy LMU University Hospital, LMU Munich Munich Germany; ^2^ Charlotte Fresenius Hochschule Munich Germany

**Keywords:** adverse childhood experiences, attachment insecurity, CBASP, persistent depressive disorder, therapeutic working alliance

## Abstract

**Background:**

Persistent depressive disorder (PDD) is marked by interpersonal dysfunction, presenting significant challenges for effective treatment. The Cognitive Behavioural Analysis System of Psychotherapy (CBASP) is specifically designed for PDD, offering a structured framework to foster a meaningful therapeutic relationship. However, its effectiveness in patients with attachment insecurities—commonly observed in PDD—remains unclear. This study examined the bidirectional relationship between the therapeutic working alliance and depressive symptoms, alongside the moderating role of attachment anxiety and attachment avoidance, in patients undergoing inpatient CBASP.

**Methods:**

A total of 164 patients completed a 10‐week inpatient CBASP programme. Depressive symptoms and the therapeutic working alliance were assessed weekly via self‐report measures, while attachment anxiety and attachment avoidance were evaluated before and after treatment. Linear mixed‐effects models were employed to analyse symptom and alliance trajectories, their reciprocal influences and the moderating effects of attachment insecurity.

**Results:**

Over the course of treatment, depressive symptoms significantly decreased, while therapeutic alliance scores significantly increased. In addition, attachment anxiety decreased significantly. Attachment anxiety and attachment avoidance did not significantly moderate changes in depressive symptoms or therapeutic working alliance over time. Increases in the working alliance predicted subsequent reductions in depressive symptoms, and improvements in symptoms predicted subsequent strengthening of the working alliance. Attachment anxiety and attachment avoidance did not moderate these reciprocal associations.

**Conclusion:**

Findings demonstrate a robust bidirectional relationship between working alliance and depressive symptomatology, independent of attachment insecurity. A brief CBASP intervention effectively reduces symptoms and strengthens the therapeutic alliance, highlighting its clinical utility in treating PDD.

## Summary

- Establishing a robust therapeutic alliance is essential and linked to improvements in depressive symptoms.
- Findings indicate that the Cognitive Behavioural Analysis System of Psychotherapy (CBASP) effectively enhances the therapeutic relationship while alleviating depressive symptoms, irrespective of patients' attachment insecurities.
- Clinicians should emphasize developing and maintaining the therapeutic relationship throughout treatment, given its pivotal, bidirectional influence on patient outcomes.

## 1 Introduction

Persistent depressive disorder (PDD) is characterized by psychosocial impairment (Angst et al. 2009; Gustavsson et al. 2011; Klein et al. 1988), high comorbidity (Köhler et al. 2019), low rates of spontaneous remission (McCullough et al. 1988) and poorer treatment outcomes compared to episodic depression (Stewart et al. 1989; Thase et al. 1994). Early onset, frequent association with adverse childhood experiences, high rates of suicidality and pervasive interpersonal dysfunction further complicate treatment (Brakemeier et al. 2021; Wolf et al. 2022, 2023). Therefore, DSM‐5 (American Psychiatric Association 2013) introduced PDD as a distinct diagnosis encompassing dysthymia with or without major depressive episodes, chronic major depression and recurrent major depression of at least 2 years' duration, including cases with severe symptoms.

The Cognitive Behavioural Analysis System of Psychotherapy (CBASP) was developed by McCullough (1984, 2000, 2006) specifically for the treatment of PDD, in response to the limited effectiveness of traditional psychotherapies for this patient group. CBASP addresses the enduring impact of early interpersonal trauma, maladaptive cognitive schemas and interpersonal dysfunction. Treatment goals include fostering social problem‐solving, increasing interpersonal empathy and modifying learned helplessness. Corrective interpersonal experiences are facilitated through disciplined personal involvement (McCullough 2012), in which therapists engage authentically but strategically to reshape maladaptive expectations and behaviours. Multiple studies have demonstrated the efficacy of CBASP in reducing symptoms of PDD (Keller et al. 2000; Negt et al. 2016; Sabaß et al. 2018; Schramm et al. 2017). Its strong interpersonal focus is particularly relevant given the high prevalence of adverse childhood experiences in this patient group (Brakemeier et al. 2018, 2021; Nanni et al. 2012; Struck et al. 2020), with evidence suggesting superior outcomes of CBASP for individuals with such experiences compared to other treatment approaches (Goerigk et al. 2024; Klein et al. 2018; Nemeroff et al. 2003; Schramm et al. 2017).

The therapeutic working alliance, defined by Bordin (1979) as the collaboration on tasks and goals within an affective bond, has been identified in several meta‐analyses as a robust predictor of psychotherapy outcomes (Horvath et al. 2011; Horvath and Symonds 1991; Lambert and Barley 2001). A strong therapeutic working alliance has been shown to facilitate the use of therapeutic techniques (Zilcha‐Mano 2017), however, the alliance itself may function as a curative factor, as building or repairing the alliance can offer a corrective experience (Hatcher 2010). In this context, Zilcha‐Mano (2017) proposed a two‐part model of the therapeutic working alliance, distinguishing between trait‐like and state‐like components. The trait‐like component reflects a patient's general capacity to form strong interpersonal relationships, enabling but not driving change itself. The state‐like component refers to alliance changes during therapy that directly foster improvement, potentially reshaping patients' internal representations of themselves and others, which is in line with theories of interpersonal relationships (such as attachment theory, Bowlby 1988), ultimately enhancing their ability to form and maintain fulfilling bonds. For patients with PDD, establishing a strong therapeutic working alliance can be challenging due to interpersonal distrust, emotional withdrawal and rigid relational patterns (McCullough 2000; Moore et al. 2003).

While the therapeutic working alliance has been traditionally examined as a unidirectional predictor of symptom change (Crits‐Christoph et al. 2011; Falkenström et al. 2013; Strunk et al. 2012; Webb et al. 2014), researchers suggest that symptom severity can also influence the quality of the therapeutic working alliance (Barber 2009; DeRubeis et al. 2005). Recent evidence points to a reciprocal relationship between the therapeutic working alliance and symptom change over the course of therapy (Beierl et al. 2021; Xu and Tracey 2015). Specifically, this was demonstrated in a study of individual therapy with clients presenting diverse concerns such as anxiety, depression, interpersonal difficulties and family issues (Xu and Tracey 2015). In this study, a stronger alliance predicted later symptom reduction and, in turn, symptom improvement predicted subsequent increases in alliance, indicating a mutually reinforcing reciprocal relationship over time. In patients with post‐traumatic stress disorder (PTSD) treated with trauma‐focused cognitive therapy, patient‐ and therapist‐rated alliance after the first session predicted end‐of‐treatment symptoms (Beierl et al. 2021). However, only therapist‐rated alliance showed a reciprocal relationship throughout the course of therapy, with higher alliance predicting lower subsequent symptoms and lower symptoms predicting higher subsequent alliance. Understanding this reciprocal interplay, as well as potentially mediating factors, is crucial for capturing the therapeutic working alliance's full clinical significance and avoiding oversimplification of therapeutic processes (Norcross and Lambert 2014).

Examining which factors moderate this reciprocal relationship is critical for tailoring therapeutic interventions. Among these factors, attachment may play a particular role, as described in the following. Attachment theory (Bowlby 1969, 1973, 1980) posits that early caregiver experiences shape internal working models that guide later relationships. Secure attachment fosters trust and effective bonding (Bowlby 1988; Cassidy and Shaver 2008), whereas insecure attachment, which is characterized by attachment anxiety (hyperactivating strategies such as dependence and heightened vigilance) or attachment avoidance (deactivating strategies such as emotional distancing and distrust) (Mikulincer and Shaver 2003), is linked to depressive symptoms (Nanni et al. 2012; Nelson et al. 2017) and interpersonal dysfunction (Bifulco et al. 2006; Whiffen et al. 1999; Widom et al. 2017). Anxious attachment appears more strongly linked to depressive symptoms than attachment avoidance (Sabaß et al. 2022; Zheng et al. 2020), and is particularly associated with persistent forms of depression (Mikulincer and Shaver 2003). Adverse childhood experiences are key determinants of insecure attachment (Baer and Martinez 2006; Cicchetti et al. 2006; Cyr et al. 2010; Sabaß et al. 2022) and are frequently reported by patients with PDD (Brakemeier et al. 2018, 2021; Nanni et al. 2012; Struck et al. 2020). These experiences encompass different forms of physical, sexual and emotional abuse, as well as physical and emotional neglect (Wingenfeld et al. 2010). Insecure attachment may undermine trust and engagement in therapy, complicating alliance formation (McCullough 2000). CBASP's interpersonal focus and therapist involvement are designed to address these challenges. Neglect may have distinct effects compared to abuse, often signalling rejection and abandonment, whereas abuse may lead to discomfort with closeness (Gauthier et al. 1996). Physical abuse has been linked to avoidant attachment (Unger and De Luca 2014), and neglect to anxious attachment (Finzi et al. 2001), although findings are mixed (Widom et al. 2017). Therapy has the potential for enhancing attachment security (Taylor et al. 2015). Changes in relational patterns with the therapist can alter internal representations of self and others (Bowlby 1988) and thereby reduce attachment insecurity.

This study aims to extend previous research by examining the reciprocal effect of the therapeutic working alliance and depressive symptomatology over a 10‐week inpatient CBASP programme. Furthermore, we investigate the moderating role of attachment anxiety and avoidance, as well as their change throughout treatment. Specifically, we hypothesized that: (1) depressive symptoms decrease, whereas therapeutic working alliance scores increase over the course of treatment; (2) attachment anxiety and attachment avoidance decrease after treatment; (3) attachment anxiety and attachment avoidance moderate the trajectories of depressive symptom reduction and therapeutic working alliance development; (4) higher scores of therapeutic working alliance are associated with lower subsequent depressive symptoms, whereas lower depressive symptoms are associated with higher subsequent therapeutic working alliance scores; and (5) attachment anxiety and avoidance moderate this reciprocal relationship.

## 2 Methods

### 2.1 Participants

Data were collected at the Department of Psychiatry and Psychotherapy, University Hospital of the Ludwig Maximilians University (LMU) Munich, Germany, between 2018 and 2024, as part of a larger naturalistic study on the effectiveness of a 10‐week CBASP inpatient psychotherapy (German Clinical Trial Register ID: DRKS00019821). The study adhered to the Declaration of Helsinki and was approved by the LMU ethics committee (EK‐No. 713‐15). A total of 178 patients entered the study; 14 were excluded due to excessive missing data (> 50%), leaving 164 patients (71 male, 93 female; age 18–66, M = 39.40, SD = 12.71). Inclusion criteria were 18–65 years in age, fluency in German and willingness to participate. All participants provided written informed consent. Exclusion criteria included a primary diagnosis of bipolar disorder, psychosis, PTSD, social phobia, panic disorder, generalized anxiety disorder, acute suicidality, pregnancy or severe somatic illness requiring primary medical care. Diagnoses were assessed using the German Structured Clinical Interview for DSM‐IV (SCID‐I); and after 2019, the SCID‐5‐CV (Beesdo‐Baum et al. 2019). The majority of patients (85.3%) met DSM‐5 criteria for PDD, with the remainder primarily presenting with recurrent or moderate to severe episodic depressions.

### 2.2 Treatment

All participants took part in a multimodal CBASP programme over the course of 10 weeks, based on the CBASP manual by McCullough (2000), which was adapted for inpatient care (Brakemeier et al. 2015, 2021). The programme included two individual (20 × 50 min) and two group therapy sessions per week (100 + 50 min), as well as weekly mindfulness training (50 min), physical therapy (50 min) and occupational therapy (100 min). Additional components comprised regular medical rounds and a weekly 25‐min nurse–patient encounter. CBASP psychotherapy was provided by two medical doctors as well as four psychologists (psychological psychotherapists or psychologists in advanced psychotherapy training) who received weekly supervision by the attending physician as well as monthly training by a member of the German CBASP network. Psychopharmacological treatment followed standardized algorithms according to national depression guidelines (Schneider et al. 2017).

### 2.3 Materials

Depressive symptoms were assessed in Week 1 and then weekly using the German version of the Beck Depression Inventory‐II (BDI‐II; Beck et al. 1996), a 21‐item self‐report measure rated on a 4‐point Likert scale (0–3) in terms of intensity. Scoring guidelines classify scores between 0–8 as no depression, 9–13 as minimal depression, 14–19 as mild depression, 20–28 as moderate depression, and 29–63 as severe depression. BDI‐II has demonstrated good psychometric properties in both clinical and nonclinical samples (Kühner et al. 2007).

The therapeutic working alliance was assessed at the end of each treatment week using the 12‐item Working Alliance Inventory—Short, patient version (WAI‐S‐P; Tracey and Kokotovic 1989), derived from the original 36‐item WAI (Horvath and Greenberg 1989). It assesses three subscales—tasks, goals and bond—on a 5‐point Likert scale (1 = *rarely*, 5 = *always*). Patients were informed that responses would not be shared with their therapist. Following common practice (Zilcha‐Mano 2017), a total score was used to indicate overall alliance strength. In both inpatient and outpatient settings, this instrument has shown good psychometric properties (Munder et al. 2010).

Attachment anxiety and avoidance were assessed at baseline and post‐treatment using the German version of the Relationship Scales Questionnaire (RSQ; Griffin and Bartholomew 1994; Steffanowski et al. 2001). The RSQ consists of 30 items rated on a 5‐point Likert scale (1 = *does not apply*, 5 = *fully applies*). A two‐dimensional structure (Roisman et al. 2007) was used, comprising eight items for attachment avoidance and five for attachment anxiety, with higher mean scores reflecting greater attachment anxiety or avoidance. The German version of the RSQ has demonstrated acceptable to good internal consistency for a clinical sample (Steffanowski et al. 2001).

### 2.4 Statistical Analysis

Analyses were conducted in RStudio (Version 4.4.1; R Core Team 2024). Item‐level missing data, assumed to be Missing at Random (MAR), were imputed using person‐mean substitution within each category. For longitudinal measures, missing values were interpolated across the 10 treatment weeks for participants with ≤ 50% missing data, based on evidence supporting this approach in datasets with stable or linear trajectories (Genolini et al. 2013). Raw values were converted to long format for longitudinal modelling. A significance level of *p* < 0.05 was applied to all analyses to determine statistical significance. Linear mixed‐effects models (LMMs) were employed to assess changes in depressive symptoms and therapeutic working alliance scores over the course of treatment (Hypothesis 1). Changes in attachment anxiety and avoidance from pre‐ to post‐treatment were analysed using paired‐samples *t*‐tests (Hypothesis 2). LMMs were further used to examine whether attachment anxiety and avoidance moderated the trajectories of symptom reduction and alliance development (Hypothesis 3), to test the reciprocal relationship between the therapeutic working alliance and depressive symptoms (Hypothesis 4) and to investigate whether attachment anxiety and avoidance moderated this reciprocal relationship (Hypothesis 5). Furthermore, analyses regarding the reciprocal effect of the therapeutic working alliance and depressive symptomatology were conducted to distinguish within‐person from between‐person effects. Disaggregation was achieved via person‐mean centering, entering both person‐specific means (between‐person effects) and deviations from these means (within‐person effects) as predictors. LMMs were estimated using the lmerTest package in R, applying Satterthwaite's approximation for degrees of freedom to compute *p*‐values.

## 3 Results

Hypothesis 1 *Depressive symptoms and the therapeutic working alliance over the course of treatment*.

**FIGURE 1:**
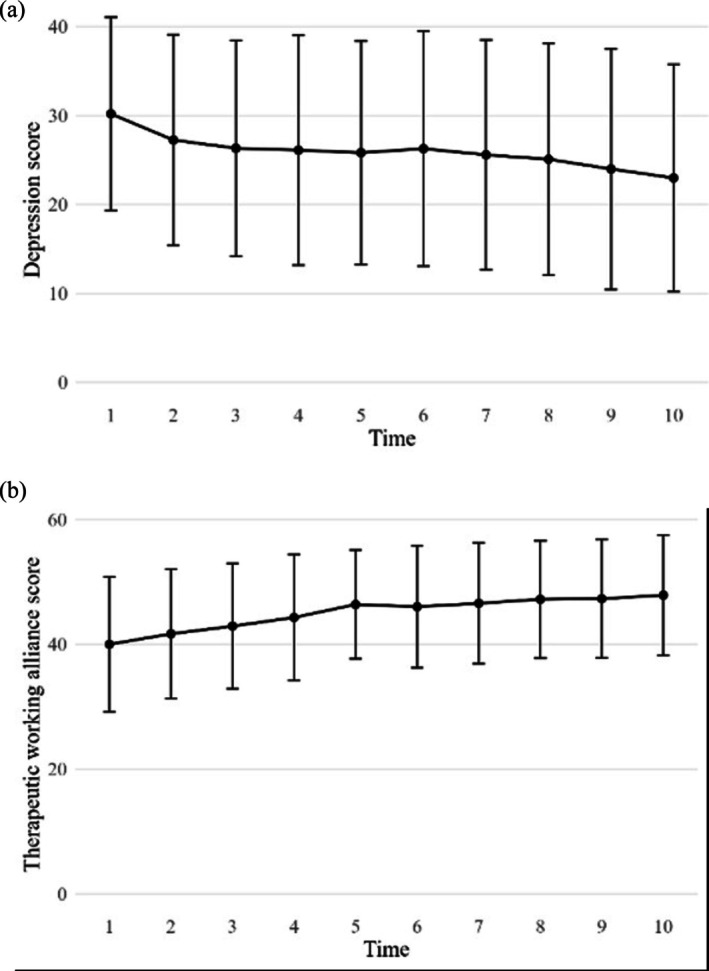
Mean levels of (a) Depression (BDI‐II) and (b) the Therapeutic Working Alliance (WAI‐S‐P) across the 10‐week CBASP Intervention. *Note:* BDI‐II: Beck Depression Inventory‐II. WAI‐S‐P: Working Alliance Inventory—Short, patient version. Mean scores of the depressive symptoms and the therapeutic working alliance across the 10 weekly assessment points. Error bars indicate one standard deviation.

Results of LMMs showed a significant decrease of depressive symptoms over the course of treatment (*b* = −0.58, SE = 0.08, *t*(163) = −7.23, *p* < 0.001), and a significant increase of the therapeutic working alliance scores (*b* = 0.84, SE = 0.09, *t*(163.03) = 9.45, *p* < 0.001). Figure 1 illustrates the visual trajectories of depressive symptoms and the therapeutic working alliance throughout the course of treatment. When comparing the beginning of treatment, participants reported a mean depression score of M = 30.20 (SD = 10.87), which decreased to M = 23.00 (SD = 12.78) by the end of treatment, indicating a shift from severe to moderate levels of depression.

Hypothesis 2 *Change in attachment anxiety and attachment avoidance*.

Paired‐samples *t*‐tests showed a significant decrease in attachment anxiety from baseline (M = 2.52, SD = 0.98) to post‐treatment (M = 2.39, SD = 0.95), *t*(128) = 2.25, *p* = 0.013, *d* = 0.20). In contrast, attachment avoidance only showed a trend towards a significant decline (M = 2.86, SD = 0.53 to M = 2.80, SD = 0.54, *t*(128) = 1.46, *p* = 0.074, *d* = 0.13).

Hypothesis 3 *Moderation of attachment anxiety and attachment avoidance on changes in depressive symptoms and the therapeutic working alliance*.

LMMs showed no significant moderation of changes in depressive symptoms over time by attachment anxiety (*b* = 0.07, SE = 0.08, *t*(162) = 0.85, *p* = 0.397), or by attachment avoidance (*b* = −0.22, SE = 0.15, *t*(162) = −1.50, *p* = 0.136), suggesting that changes in depressive symptoms over time did not differ significantly by the level of attachment anxiety or avoidance.

Similarly, attachment anxiety (*b* = 0.15, SE = 0.09, *t*(162) = 1.74, *p* = 0.084), and attachment avoidance (*b* = 0.13, SE = 0.17, *t*(161.98) = 0.75, *p* = 0.452) did not significantly moderate the development of the therapeutic working alliance over time, suggesting that changes in the therapeutic working alliance were not influenced by levels of attachment anxiety.

Hypothesis 4 *Reciprocal effect of changes in depressive symptoms and therapeutic working alliance*.

LMMs showed a significant negative effect of the therapeutic working alliance on subsequent depressive symptoms (*b* = −0.10, SE = 0.02, *t*(1406.69) = −4.57, *p* < 0.001, *β* = −0.08, 95% CI [−0.12, −0.05]). This suggests that higher alliance scores were associated with lower depressive symptoms at the subsequent time point. An LMM separating within‐ and between‐person effects showed a significant within‐person effect (*b* = −0.09, SE = 0.02, *t*(1472) = −4.27, *p* < 0.001, *β* = −0.12, 95% CI [−0.17, −0.06]), indicating that patients experienced lower depressive symptoms when they reported stronger therapeutic working alliance scores than their own average at the previous measurement point. The between‐person effect was not significant (*b* = −0.00, SE = 0.01, *t*(1472) = −0.23, *p* = 0.819), indicating that individuals with generally higher alliance scores did not show lower overall subsequent depressive symptoms.

Vice versa, an LMM showed that lower depressive symptoms predicted higher subsequent alliance scores (*b* = −0.11, SE = 0.03, *t*(1335.53) = −4.33, *p* < 0.001, *β* = −0.14, 95% CI [−0.21, −0.08]). This suggests that lower levels of depressive symptoms were associated with higher subsequent ratings of the therapeutic working alliance. Results indicate a small‐to‐moderate negative association and a marginally stronger effect than the reverse direction based on standardized coefficients. An LMM separating within‐ and between‐person variance showed a significant within‐person effect. Therapeutic working alliance scores were higher when depressive symptoms were below a patient's own average at the prior time point (*b* = −0.10, SE = 0.03, *t*(1472) = −3.74, *p* < 0.001, *β* = −0.09, 95% CI [−0.14, −0.05]). The between‐person effect was non‐significant (*b* = −0.00, SE = 0.01, *t*(1472.00) = −0.06, *p* = 0.953), indicating that individuals with generally lower depression scores did not differ significantly in overall subsequent alliance levels.

Hypothesis 5 *Attachment anxiety and attachment avoidance as moderators for the reciprocal effect*.

All LMMs testing attachment anxiety and attachment avoidance as moderators of the bidirectional association between the therapeutic working alliance and depressive symptoms were non‐significant, with interaction effects ranging from *b* = −0.05 to 0.05, *p* = 0.088–0.961, indicating no moderating effects of attachment on either direction of the relationship.

## 4 Discussion

Our study found that weekly assessed self‐reported depressive symptoms decreased significantly from severe to moderate, and therapeutic working alliance scores increased significantly over the course of a 10‐week structured inpatient CBASP programme for patients with PDD. These findings support the effectiveness of CBASP as a treatment specifically developed for PDD (McCullough 1984) and align with previous research (Keller et al. 2000; Negt et al. 2016; Sabaß et al. 2018; Schramm et al. 2017). In addition, the weekly alliance improvement reflects state‐like changes described in Zilcha‐Mano's (2017) model, potentially fostering corrective interpersonal experiences (Bowlby 1988; Zilcha‐Mano et al. 2016).

Our results further support the assumption of a reciprocal relationship between the therapeutic working alliance and depressive symptoms during treatment. Higher alliance scores predicted lower subsequent depressive symptoms, and lower symptoms predicted stronger subsequent alliance ratings. When distinguishing within‐ from between‐person variance, both directions showed significant within‐person effects, suggesting that patients experienced symptom improvement when their alliance ratings exceeded their own average and, conversely, reported stronger alliances when their symptoms were lower than their usual one's. Between‐person effects were non‐significant, indicating that overall higher average alliance or symptom levels did not predict each other. These findings align with previous research and meta‐analyses highlighting the alliance as a robust predictor of psychotherapy outcomes (Crits‐Christoph et al. 2011; Falkenström et al. 2013; Horvath et al. 2011; Horvath and Symonds 1991; Lambert and Barley 2001; Strunk et al. 2012; Webb et al. 2014), while also supporting the assumption that symptom severity can influence alliance development (Barber 2009; DeRubeis et al. 2005). The bidirectional pattern underscores the dynamic interplay between relational and symptomatic change (Beierl et al. 2021; Xu and Tracey 2015), pointing to a potential benefit for therapists in monitoring simultaneous progressions, as changes in either may signal the need for therapeutic adjustments.

Contrasting our hypotheses, neither attachment anxiety nor attachment avoidance significantly moderated changes in depressive symptoms or the therapeutic working alliance over the course of treatment, and no effects emerged for either direction of the reciprocal association between depressive symptomatology and the therapeutic working alliance. These findings contrast with prior research linking attachment insecurity to overall vulnerability for depression (Nanni et al. 2012; Nelson et al. 2017), and to difficulties in forming close relationships including with therapists (Bifulco et al. 2006; Bowlby 1973; Diener et al. 2009; Scott et al. 2012; Whiffen et al. 1999; Widom et al. 2017). Anxious attachment, which is associated with negative self‐image, hypersensitivity to rejection and emotional reactivity (Sabaß et al. 2022; Zheng et al. 2020), and avoidant attachment, marked by emotional distancing and suppression of needs (Mikulincer and Shaver 2003), were both expected to inhibit emotional engagement and alliance formation. Across both domains, the absence of moderation effects may reflect the influence of CBASP's interpersonal focus, which is designed to foster corrective relational experiences (McCullough 2012). This focus is thought to reduce typical alliance barriers associated with insecure attachment, such as abandonment fears in anxiously attached patients, as well as perceived closeness threats in individuals with avoidant attachment. However, a further explanation may be methodological: attachment insecurity was assessed globally rather than in relation to the therapeutic relationship, despite evidence that attachment orientations vary across relational contexts and that relationship‐specific measures may better predict therapeutic processes (Baldwin et al. 1996; Fraley et al. 2011). Taken together, these findings suggest that inpatient CBASP may help overcome alliance barriers and reduce depressive symptoms even in patients with attachment insecurity, supporting its effectiveness in this population.

Finally, we observed that patients showed reduced levels of attachment anxiety after completing the inpatient CBASP treatment programme. Attachment anxiety significantly decreased from baseline to post‐treatment, while attachment avoidance showed a trendwise decrease. These results suggest that even within a relatively short, 10‐week treatment period, CBASP may already begin to positively influence patients' broader internal working models of self and others (Bowlby 1988). As the therapeutic relationship in CBASP aims to provide corrective interpersonal experiences (McCullough 2012), anxiously attached patients may begin to perceive social interactions in less anxiety‐inducing ways. In contrast, avoidant attachment is characterized by emotional distancing and discomfort with closeness (Mikulincer and Shaver 2003), which are traits that may require longer or more intensive intervention to shift meaningfully. While CBASP's interpersonal structure might begin to challenge avoidant tendencies, the treatment duration may not be sufficient for measurable effects. These findings add to evidence that psychotherapy, including CBASP, may enhance attachment security (Taylor et al. 2015).

### 4.1 Practical Implications

This study highlights key clinical implications for the treatment with CBASP. Findings support a bidirectional relationship between the therapeutic working alliance and depressive symptoms, underscoring the value of regularly monitoring both throughout treatment to guide tailored interventions. Significant symptom reduction and alliance strengthening following inpatient CBASP confirm its effectiveness across patients with varying levels of attachment insecurity. Moreover, modest decreases in attachment anxiety and avoidance indicate that interpersonal change is possible even in relatively brief inpatient CBASP formats. However, individuals with higher levels of attachment insecurity might benefit from longer or more intensive treatment formats, a question that warrants further investigation.

### 4.2 Strength and Limitations

A major strength of this study is its longitudinal design with weekly self‐reported assessments of depressive symptoms and therapeutic working alliance scores over a 10‐week inpatient CBASP programme. This longitudinal measurement enabled a detailed examination of dynamic therapeutic processes and the application of within‐ and between‐person modelling via advanced statistics. Moreover, conducting the study in a naturalistic inpatient setting further enhances its ecological validity.

However, there are certain limitations that need to be considered. Limitations include the exclusive use of self‐report measures, which are subject to social desirability, response bias and memory. Other common therapeutic factors (e.g., patient characteristics, therapist allegiance or confidence in their therapeutic approach, and client expectations) were not assessed and could have influenced outcomes (Wampold 2001). The attachment measure captured global patterns rather than therapy‐specific dynamics, potentially limiting detection of moderating effects. Without a control group, causal inferences about CBASP's specificity remain tentative, and the absence of follow‐ups precludes conclusions about long‐term effects.

Future research should use relationship‐specific attachment measures to better capture experiences within the therapeutic dyad. Moreover, it may be beneficial to assess the therapeutic working alliance from both patient and therapist perspectives, as these may differ and provide complementary insights. Follow‐up assessments are needed to examine the durability of effects, and control groups should be included to test the specificity of CBASP‐related changes. Differentiating alliance components may clarify mechanisms of symptom change.

## 5 Conclusion

This study supports the assumption of a bidirectional relationship between the therapeutic working alliance and depressive symptoms in patients with PDD undergoing inpatient CBASP treatment. Within‐person increases in alliance predicted subsequent symptom reduction, and symptom improvement predicted stronger subsequent alliance scores. Depressive symptoms decreased and alliance scores increased significantly over treatment, alongside a significant reduction in attachment anxiety. Of note, neither attachment anxiety nor attachment avoidance moderated alliance‐symptom associations. Overall, our findings underscore the potential for CBASP to foster interpersonal improvement, symptom reduction and enhanced therapeutic working alliance even within brief inpatient formats.

## Funding

The authors have nothing to report.

## Ethics Statement

This study was approved by the LMU ethics committee, Munich, Germany, Approval Number [713‐15]. All procedures performed were in accordance with the ethical standards of the institutional research committee and with the 1964 Helsinki Declaration and its later amendments.

## Consent

Informed consent was obtained from all individual participants included in the study.

## Conflicts of Interest

The authors declare no conflicts of interest.

## Data Availability

The data that support the findings of this study are available from the corresponding author upon reasonable request.
